# Scented grasses in Norway—identity and uses

**DOI:** 10.1186/s13002-015-0070-y

**Published:** 2015-12-23

**Authors:** Torbjørn Alm

**Affiliations:** Tromsø museum, University of Tromsø, PO Box 6050, Langnes, N-9037 Tromsø, Norway

**Keywords:** *Anthoxanthum nipponicum*, *Hierochloë odorata*, *Milium effusum*, Braids, Perfume

## Abstract

**Background:**

Some grass species are richer in coumarin and thus more sweetly scented than others. These have been eagerly sought after in parts of Norway, but the tradition has been weakly documented, both in terms of the species collected, their vernacular names, and uses.

**Methods:**

Based on literature data and a substantial body of information collected during my own ethnobotanical field work, artefacts and voucher specimens, the grass species are identified, and their uses clarified.

**Results:**

In Norwegian literature, the tradition of collecting and using scented grasses has received little attention, and past authors largely refer it to *Anthoxanthum* spp. The tradition’s concentration to the Sámi strongholds of northernmost Norway, and most authors’ lacking knowledge of the Sámi language, have contributed to the weak and misleading coverage in previous publications. Coumarin-rich grass species are well known in folk tradition in northernmost Norway, as *luktegress* (Norwegian, “scent grass”), *háissasuoidni* (North Sámi, “scent grass”), *hajuheinä* (Finnish, “scent grass”), or similar terms. They have been (and still are) frequently collected, and used as perfume, for storing with clothes, and a number of other purposes. Despite literature records identifying the species used as *Anthoxanthum odoratum* coll. (including *A. nipponicum),* the main source utilized in North Norway is *Hierochloë odorata*, both ssp. *arctica* and ssp. *odorata. Anthoxanthum nipponicum* and *Milium effusum* are alternative, but infrequently used sources of material, depending on local tradition and availability.

**Conclusion:**

By far the most important grass species hiding behind the “scented grass” tradition in Norway is *Hierochloë odorata. Anthoxanthum nipponicum* is also used, but much less frequently, and only a single record confirms the use of *Milium effusum*. Only the foliage of *Hierochloë* provides suitable material for making traditional braids. The three major ethnic groups in Norway have all utilized scented grasses as perfume and for storing with clothes, but the tradition’s geographical concentration to the far north of Norway (Finnmark and NE Troms), suggests that it has originally mainly been a Sámi tradition, adopted by their neighbours.

## Background

Scented or coumarin-rich grasses have found a variety of uses in the northern hemisphere, mainly on account of their sweet and pleasant scent – as perfume for people and dwellings, but also to flavour food and drink, e.g. the well-known *żubrówka* of Poland, in folk medicine, and, at least among the indigenous tribes of North America, in various religious rituals. It is, however, not my intention to provide a global review of scented grasses – a daunting task even for Europe, given the paucity of accounts available, and certainly so in the major western languages.

**Fig. 1:**
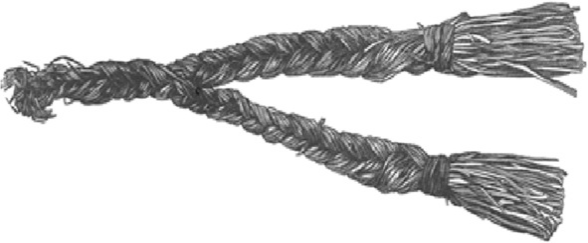
A North Sámi braid of scented grass as depicted by F.C. Schübeler in 1886 ([2]: 259), wrongly identified as deriving from *Anthoxanthum odoratum* L

In the northernmost part of Norway, sweetly scented grasses constitute a well established part of folk tradition. Grass material intended for perfume and similar purposes has been collected at numerous sites. Despite this, such grasses have received little attention in Norwegian ethnobotanical literature. The first, brief comment was made by Johan Ernst Gunnerus in 1772 ([1]: 117), who noted *marigras* (“Mary’s grass”) and *lugtgrass* (“scent-grass”) as Norwegian vernacular names for *Holcus odoratus,* i.e. *Hierochloë odorata* (L.) Wahlenb. A more extensive comment is provided by Fredrik Christian Schübeler in 1886 ([2]: 259), who noted their main uses, and depicted a North Sámi grass braid (reproduced in Fig. 1), according to him made of *Anthoxanthum odoratum* L. coll. Just Qvigstad ([3]: 59, cf. [4]: 305) and Kristian Nissen ([5]: 2–3), commenting on Sámi tradition, suggested that both *Anthoxanthum odoratum* L. (coll.) and *Hierochloë odorata* provided source material. Ove Arbo Høeg ([6]: 224) and Olav Johansen [7] identified the species used as *Anthoxanthum odoratum,* although the former, in his vast collection of Norwegian plant lore, noted that *Hierochloë odorata* could also be used, at least locally; an example is given from Beitstad in Trøndelag, central Norway. No voucher specimens are cited in any of these works. In his compilation of Sámi folk medicine, Adolf Steen [8] has a brief entry on such grasses. It is listed among cures utilizing *starr* (the Norwegian term for *Carex* spp.), probably mislead by the fact that both are regarded as *suoinnit* (singular *suoidni*)*, “*graminoids”, in Sámi terminology.

To my experience, the brief notes so far published on scented grasses in Norway are incomplete and, in terms of identification, partly incorrect. As noted for Swedish material by Ingvar Svanberg ([9]: 244), it is difficult or impossible to identify the species hiding behind the comments on such grasses and their uses in literature and archival sources. Voucher specimens, or specific question aimed at clarifying important characteristics, are needed to ascertain which species were used. This paper aims at doing so for Norway, and also provides substantial new material related to the uses of such grasses in both Norwegian, Sámi and Finnish tradition, i.e. among the three ethnic groups with a long history in Norway.

## Methods

This paper is based on literature data extracted from my database of more than 7000 publications containing information on plant names and plant uses in Norway, and material from my own vast collection of ethnobotanical data, based on numerous interviews, correspondence etc. The latter are referred to by the acronym EBATA, year and record number, e.g. EBATA 1992:11.

“Scented grasses” are characterized by the strong odour of coumarin, a sweet-smelling benzopyrone. Coumarins in general are found, usually as glycosides, in many other plants as well ([10]: 385, [11]: 462, [12]), ranging from woodruff (*Galium odoratum* L.) and numerous Apiaceae species to cassia (*Cinnamomum aromaticum* Nees) – the source of most of the cheap “cinnamon” on the market. Sweet clover (*Melilotus officinalis* (L.) Lam.) is a typical example, where coumarin is formed enzymatically (in this case from melilotoside) when the tissue dries and breaks down ([12]: 203]. In other words: when plant material (e.g. grasses) dries, the fragrance is released, providing newly scythed hay with its characteristic, sweet scent.

If present in quantity, coumarin provides the plants with a bitter taste, repelling insects and grazing animals. It is modestly toxic to humans ([13]: 444ff), liver and kidneys in particular, and more so to grazing animals. If plant material (e.g., hay) is not properly dried, dicoumaroul may form. It is chemically similar to vitamin K, and of particular concern due to its anticoagulant effect, which may cause hemorrhagic disease ([12]: 203, [14]: 910).

Although common in grasses (Poaceae), some genera and species are much richer in coumarin, and thus more fragrant, than others. Among the grasses found in Norway, four genera with eight species and subspecies may provide suitable material for utility purposes:

(1–2) *Anthoxanthum,* with two species, *A. odoratum* L. and *A. nipponicum* Honda (syn. *A. alpinum* Á. & D.Löve, *A. odoratum* L. ssp. *alpinum* (Á. & D.Löve) B.M.G.Jones & Melderis). Both are rather small, usually some 20 to 30 cm tall, and look very similar to the untrained eye. Spikes are usually denser in *A. odoratum*, and distinctly pubescent, with scattered hairs on the pedicels. The interior glumes are covered with tiny spikes, and thus appear dull. *A. nipponicum* is almost glabrous, with shiny glumes. In Norway, the two taxa are easily separated [15], although hybrids are known to occur. *A. odoratum* is a lowland plant, extending northwards along the coast to the southernmost part of Troms in northern Norway; *A. nipponicum* is common in the mountains and southwards along the coast to Central Norway [15, 16].

(3–6) *Hierochloë*, with four species and subspecies: *H. alpina* (Sw.) Roem. & Schult., *H. hirta* (Schrank) Borbás, *H. odorata* (L.) Wahlenb. ssp. *odorata*, and *H. odorata* ssp. *arctica* (J.Presl.) Tzvelev. In Scandinavian floras, the latter has usually been considered a subspecies of *H. hirta,* following the treatment of Weimarck [17, 18]. In the recent Panarctic Flora treatment [19], it has been transferred to *H. odorata*, in accordance with the view of Russian botanists. In Norway, *H. hirta* is only known from the southeast [20], whereas *H. odorata* ssp. *odorata* is found throughout the country, and ssp. *arctica* is common in alpine areas and in most of central and northern Norway [21]. All three grow in the same kind of environment – damp meadows, mires and along river banks. They are almost identical to the untrained eye; even botanists find them hard to tell apart. Contrary to *Anthoxanthum*, *Hierochloë* stands are usually predominated by the long, shiny leaves, which may form dense swards. *H. alpina* is found only in dry, alpine habitats, and rarely in such profusion that it would allow much in terms of collecting material for utility purposes.

It is worth noting that *Anthoxanthum* and *Hierochloë* are closely related [22, 23]. Although the European species of either look distinctly different, some Asian species bridge the gap.

(7) *Cinna latifolia* (Trevir. ex Göpp.) Griseb., which is an infrequent species in Norway (see map and discussion in [24]), and certainly so in the north, where it has only been confirmed from two localities, in Hattfjelldal, Nordland, and in Alta, Finnmark.

(8) *Milium effusum* L., which is common throughout Norway, growing in nutrient-rich forests and slopes ([16]: 1035–1036). It is often more than 1 m tall, with broad, short leaves and stout culms.

## Results

### Identity

With the above-mentioned taxa in mind, it may be assumed that whenever the records mention braids made from long and flexible leaves, *Hierochloë odorata* has been used. It is possible to make braids from the culms of *Anthoxanthum* and *Milium effusum*, but I have never seen any such artefacts. However, a few records clearly state that straws were used, and still mention braids, which may point to just such deviant items – or areas were both *Hierochloë* leaves and *Anthoxanthum* and/or *Milium* culms were collected, and possibly only the former used for braids.

**Fig. 2:**
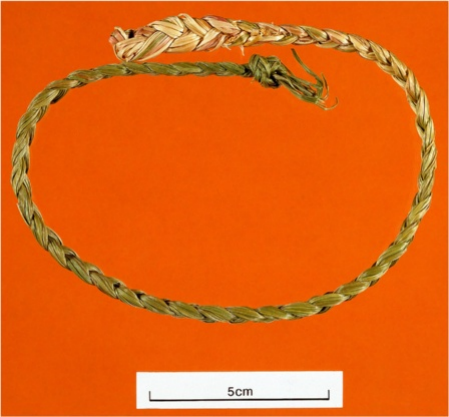
Photograph of a similar modern Skolt Sámi braid of *Hierochloë odorata* from Neiden in Sør-Varanger, Finnmark (voucher: ESTA 2001:1). Photograph by Adnan Igacic, Tromsø museum

**Fig. 3:**
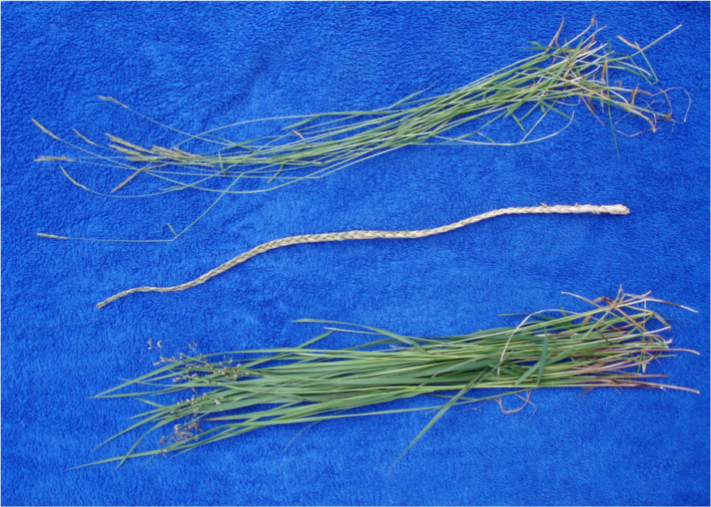
A scented grass braid (middle) compared to the main sources of suitable plant material available in northern Norway: *Anthoxanthum nipponicum* (at top), where only the culms are of suitable length, and *Hierochloë odorata*, with long, flexible leaves that provide the necessary material for braiding (bottom). Photograph: Torbjørn Alm 2006

As noted in the introduction, previous Norwegian authors have generally referred the grass(es) collected and used for their fine scent to *Anthoxanthum odoratum* coll. (including *A. nipponicum*). This may be correct as far as southern Norway is concerned, where *Anthoxanthum* spp. has some reputation both as a “perfumed” grass and for various other purposes [6]. Unfortunately, only a single voucher specimen confirms this (from Forsand in Rogaland, related to Ove Arbo Høeg’s record no. 567 (cf. [6]: 224), with voucher in TROM). Thus, identifications are generally based on interviews and other oral or written ethnobotanical imformation, and perhaps coloured by the rather bold assertion by F.C. Schübeler ([2]: 259) that all such scented grasses may be identified as *Anthoxanthum odoratum*. However, the North Sámi “scented grass” braid depicted by Schübeler, reproduced in Fig. 1, cannot derive from *Anthoxanthum*; the long leaves needed for making such braids are typical of *Hierochloë odorata* (Figs. 2 and 3). This is confirmed by recent ethnobotanical investigations in the far north of Norway (Table 1).

**Table 1:** Identity of material collected as “scented grass” in Norway – based on voucher specimens from traditional collecting sites, material shown to and confirmed by informants, or braids made from the long leaves of *Hierochloë* spp

Taxon / site	Ethnic origin	Type of material, voucher	Further information
*Anthoxanthum nipponicum*			
Troms: Kvænangen: Løkeng	North Sámi	Voucher specimen (ESTA 2012:1)	EBATA 2010:47, 2012:5, 2012:7
*Hierochloë odorata*			
Troms: Kvænangen: Burfjord	North Sami	Detailed description	EBATA 2012:5, 2012:8
Finnmark	North Sámi	Drawing of braid	[2]: 259
Finnmark: Guovdageaidnu/Kautokeino: Suolovuopmi	North Sámi	Braid prepared for sale (ESTA 2006:43)	EBATA 2006:126
Finnmark: Lebesby: Kunes	North Sámi	Detailed description	EBATA 2015:24
Finnmark: Båtsfjord: Syltefjord	Norwegian	Voucher specimen (ESTA 2006:39)	EBATA 2006:117
Finnmark: Unjárga/Nesseby	North Sámi	Braid at Varanger samiske museum	[25]: 66
Finnmark: Unjárga/Nesseby: Bergebydalen	North Sami	Braid (ESTA 2007:2)	
Finnmark: Unjárga/Nesseby: Bergebydalen	North Sámi	Bundle of leaves collected for braiding (ESTA 2007:17)	
Finnmark: Unjárga/Nesseby	North Sámi	Two braids (ESTA 2009:4)	
*Hierochloë odorata* ssp. *arctica*			
Finnmark: Alta: Seiland: Hakkstabben	North Sámi	Voucher specimena (ESTA 2015:1; TROM V-968141)	EBATA 2015:13
Finnmark: Kvalsund: Beritsjord	North Sámi	Traditional site; voucher specimens (ESTA 2006:11, TROM V-962258)	EBATA 2006:82
Finnmark: Guovdageaidnu/Kautokeino: Heammonjávvi	North Sámi	Traditional site; voucher specimen (TROM V-54979)	EBATA 1996:10
Finnmark: Vadsø: Skallelv	Finnish	Tradiitional site; voucher specimens (ESTA 2006:15, 21, 26)	EBATA 2006:106, 111, 1112, 113, 114, 115
Finnmark: Sør-Varanger: Neiden	Skolt Sámi	Traditional site; voucher specimen (TROM V-86071)	
Finnmark: Sør-Varanger: Neiden	Skolt Sámi	Braid (ESTA 2001:1)	
*Hierochloë odorata* ssp. *odorata*			
Finnmark: Kvalsund: Gjevebukta	North Sámi	Traditional site; voucher specimens (ESTA 2006:12, 13; TROM V-962264)	EBATA 2006:87
Finnmark: Vadsø: Ekkerøy	Norwegian	Traditional site; voucher specimen (ESTA 2006:40)	EBATA 2006:118,121,122;
*Milium effusum* L.			
Finnmark: Nordkapp: Repvåg	Norwegian	Traditional site, voucher specimen (ESTA 2006:2)	EBATA 2006:38, 46, 50

It may be noted here that Schübeler ([2]: 259) “explained” the more extensive use of what he interpreted as *Anthoxanthum odoratum* coll. in northernmost Norway by stating that “In Finmarken, where this grass has a stronger aroma than in the southern parts of the country …” To my experience, there is nothing to suggest that *Anthoxanthum* (or *Hierchloë odorata*) smells more strongly in the north.

**Fig. 4:**
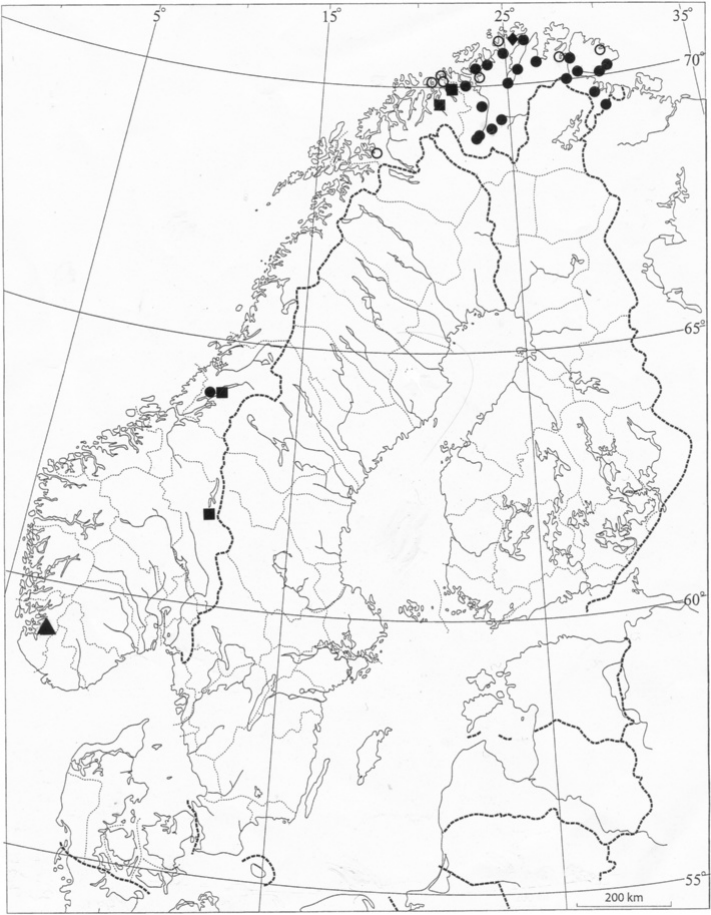
Map showing the distribution within Norway of the «scented grass” tradition, and the grass species utilized: *Anthoxanthum nipponicum* (■), *Anthoxanthum odoratum* (▲), *Hierochloë odorata* (●), *Milium effusum* (♦), and unidentified grasses, probably *Hierochloë odorata* (*○*)

Most, but not all, voucher specimens and traditional harvesting sites for “scented grasses” studied by me belong to *Hierochloë odorata.* It provides the best and preferred material for traditional braids, and certainly so in Sámi tradition. Other species have only found restricted and local use. Available material reveals the following taxa (Table 1, Fig. 4):

I) *Hierochloë odorata* ssp. *arctica*. This is the most frequently encountered taxon, especially in inland areas, where it may grow abundantly on damp meadows along the larger rivers. Some of the traditional collecting sites inspected have been in just this kind of environment. It is a frequent source of material for Sámi braids.
II) *Hierochloë odorata* ssp. *odorata*. The nominate subspecies is somewhat less frequent in my material, but has been used and collected just as ssp. *arctica*, depending on what is locally available.
III) * Anthoxanthum odoratum.* The only record referable to this species derives from southwesternmost Norway, as shown by the voucher specimen (in TROM) of Ove Arbo Høeg’s record from Forsand in Rogaland (NFS 184).
IV) * Anthoxanthum nipponicum*. Local use has been confirmed by a few, scattered records (Fig. 4). The grass collected in the Snefjord area of Måsøy, Finnmark, probably also belonged to this species, as suggested by the fact that it was not braided, and the description given: “No, it was an ordinary grass straw, it had no leaves” (EBATA 2006:89); “that fine-scent grass has no leaves” (EBATA 2006:90).
V) *Milium effusum* has only been confirmed from a single station, at Repvåg in Nordkapp, Finnmark.

As expected, the rare *Cinna latifolia* is not used. I also consider it unlikely that *Hierochloë alpina* has found any use. For obscure reasons, some (non-botanist) authors claim that this is one of the species used [25], or the only species used ([26]: 23), which would suggest that they have never seen it. It is generally infrequent, mostly found at high altitudes (low- to mid-alpine), growing on exposed ridges, where its small tufts are so depauperate that it is difficult to extract any fresh foliage from it all, and certainly not in sufficient quantity to make braids – which would be very small on account of the short and narrow leaves. In Guovdageaidnu/Kautokeino, Myrdene Anderson noted that people collected scented grasses both on moist meadows and on “dry, well-drained ground, often beneath juniper bushes” ([27]: 518). She assumed that the latter could be *Hierochloë alpina*, but *Anthoxanthum nipponicum* is a much more likely candidate. There are no upright juniper bushes in the alpine habitats of *Hierochloë alpina,* only the prostrate *Juniperus communis* L. ssp. *alpina* (Sm.) Čelak. There is no voucher (her material deposited in TROM) to accompany the record.

### Vernacular names

The three major ethnic groups in Norway have all coined vernacular names for scented grasses. In accordance with the pattern of use (see below), North Sámi has the most extensive set of names (Table 2).

**Table 2:** Vernacular names recorded for scented grasses in Norway

Ethnic origin	Translation	Locality	Identity	Source of information
Norwegian				
Duftgress	fragrant grass	Finnmark: Alta	Unidentified	EBATA 2006:6
Duftegress	fragrant grass	Finnmark: Lebesby	*Hierochloë odorata*	EBATA 2015:24
Frengras	strongly scented grass	Nord-Trøndelag: Steinkjer	*Anthoxanthum, Hierochloë*	[6]: 224
Godluktgress	fine scent grass	Troms: Nordreisa	Unidentified	EBATA 2006:88; 2015:26
Godluktgress	fine scent grass	Finnmark: Porsanger	Unidentified	EBATA 2006:93
Godluktgress	fine scent grass	Finnmark: Måsøy	?*Anthoxanthum nipponicum*	EBATA 2006:90
Godluktgress	fine scent grass	Finnmark: Båtsfjord	*Hierochloë odorata*	EBATA 2006:117
Godluktgress	fine scent grass	Finnmark: Vadsø: Vestre Jakobselv	Hierochloë odorata*	[48]: 8-9
Godluktgress	fine scent grass	Finnmark: Vadsø	Hierochloë odorata*	[54]: 44
Godluktsenna	fine scent graminoid	Finnmark: Porsanger: Leirpollen	Hierochloë odorata*	[52]: 17
Lingress	flax grass	Troms: Kvænangen	Unidentified	EBATA 2004:7
Luftgrasbonk	scent grass bundle	Hedmark: Engerdal	mainly *Anthoxanthum nipponicum*	[6]: 224; EBATA 2015:6, 2015:7
Luktegras	scent grass	Troms: Skjervøy	Unidentified	EBATA 2005:45
Luktegress	scent grass	Finnmark: Alta	*Hierochloë odorata*	EBATA 2015:13, 2015:20. 2015:21
Luktegress	scent grass	Finnmark: Kvalsund	*Hierochloë odorata*	EBATA 2006:82
Luktegress	scent grass	Finnmark: Deatnu/Tana	Hierochloë odorata*	EBATA 2006:13
Luktegress	scent grass	Finnmark: Vadsø	*Hierochloë odorata*	EBATA 2006:106,.118,121,122
Luktegress	scent grass	Finnmark: Unjárga/Nessbey	*Hierochloë odorata*	EBATA 2007:68
Luktegress	scent grass	Finnmark: Sør-Varanger	Hierochloë odorata*	EBATA 2015:27
Mixed Norwegian/Sámi names				
Haisenna	scent graminoid	Finnmark: Alta	Hierochloë odorata*	EBATA 1998:3
Haisennagress	scent graminoid grass	Finnmark: Nordkapp	*Milium effusum*	EBATA 2006:38, 46
North Sámi				
Háissasuoidni	scent grass	Finnmark: Guovdageaidnu/Kautokeino	Hierochloë odorata*	EBATA 2015:3
Háissasuoinnet	scent grasses	Finnmark: Karášjohka/Karasjok	Unidentified	EBATA 1992:11
Háissasuoinnit	scent grasses	Finnmark: Guovdageaidnu/Kautokeino	*Hierochloë odorata*	[27]: 518
Háissasuoinnit	scent grasses	Finnmark: Karášjohka/Karasjok	Unidentified	EBATA 2006:27
Háissorássi	scent grass	Finnmark: Kvalsund	Hierochloë odorata*	EBATA 2006:92
Háisuoidni	scent grass	Troms: Lavangen	Unidentified	EBATA 2006:8
Háisuoidni	scent grass	Finnmark: Guovdageaindu/Kautokeino	*Hierochloë odorata* ssp. *arctica*	EBATA 1996:10
Háisuoidni	scent grass	Finnmark: Porsanger	*Hierochloë odorata* ssp. *arctica*	EBATA 2001:4
Háisuoinnit	scent grasses	Finnmark: Guovdageaidnu/Kautokeino	*Hierochloë odorata*	[27]: 518; EBATA 2006:66
Háisuoinnit	scent grasses	Finnmark: Guovdageaidnu/Kautokeino	*Hierochloë odorata*	EBATA 2006:23
Njálggaháisuoidni	pleasant scent grass	Finnmark: Deatnu/Tana. Polmak	Hierochloë odorata*	EBATA 1996:2
Njálggarásse	pleasant grass	Finnmark: Måsøy	?*Anthoxanthum nipponicum*	EBATA 2006:89
Njálggasuoidne	pleasant grass	Finnmark: Unjárga/Nesseby	Hierochloë odorata*	[25]: 66
Njálggasuoidni	pleasant grass	Finnmark: Deatnu/Tana: Polmak	Hierochloë odorata*	EBATA 1996:2
Njálggesuoidni	pleasant grass	Finnmark: Unjárga/Nesseby	?*Hierochloë odorata*	EBATA 1996:3
Njálggsuoidne, njálggsuoidni	pleasant grass	Finnmark: Unjárga/Nesseby	*Hierochloë odorata*	EBATA 2007:67, 69, 70
Finnish				
Haisuheinät	scent grasses	Finnmark: Porsanger: Børselv	Hierochloë odorata*	[49]: 99
Hajuheinä	scent grass	Finnmark: Vadsø: Vestre Jakobselv	Hierochloë odorata*	[48]: 8
Hajuheinä	scent grass	Finnmark: Vadsø: Golnes	Hierochloë odorata*	EBATA 2006:111
Hajuheinä	scent grass	Finnmark: Vadsø: Skallnes	Hierochloë odorata*	EBATA 2006:112
Hajuheinä	scent grass	Finnmark: Vadsø: Skallelv	Hierochloë odorata*	EBATA 2006:10, 113, 114, 115
Hyvähajuheinä	fine scent grass	Finnmark: Vadsø: Skallelv	*Hierochloë odorata*	EBATA 2006:114

In some cases, the identification as *Hierochloë odorata (*marked with a*) is based on information that the leaves were braided (see text); no plants were seen. For voucher specimens, see Table 1

#### Norwegian

In the north, scented grasses are mostly called *luktegress* (“scent grass”) or *godluktgress* (“fine scent grass”). In those cases where plant material has been seen, and voucher specimens obtained, the names mostly refer to *Hierochloë odorata. Haisenna,* used for *Hierochloë odorata* in Alta, and *haisennagress* for *Milium effusum* in Nordkapp, Finnmark, are Norwegian adaptions of the Sámi *háisuoidni,* preserving the prefix, and replacing *–suoidni* with *–senna,* a widespread Norwegian term for graminoids, including *Carex* spp. and various grass species.

Ove Arbo Høeg ([6]: 224) recorded a couple of deviant Norwegian names from areas further south: *frengras* (“strongly scented grass”) from Steinkjer in Nord-Trøndelag, used for both *Anthoxanthum* and *Hierochloë*, and *luftgrasbonk* (“scent grass bundle”) from Engerdal in Hedmark, the latter supposedly for *Anthoxanthum*.

#### Sámi

The Sámi vernacular names recorded in Table 2 are all North Sámi, thus belonging to the largest of the six main Sámi languages [28]. They follow the same pattern as the Norwegian names, referring to *háisuoidni, háissasuoidni* or *háisǫsuoidni* (“scent grass”), locally replaced with the more or less synonymous *hádja* or *hágja-* (“scent”), or *njálggasuoidni* (“pleasant grass”, “well-scented grass”; *njálgga* may mean both “pleasant”, “sweet”, “well-scented”, and “well-tasting”). A combination of these may also occur: *njálggaháisuoidni* (“pleasant scent grass”), in various dialectal forms. Konrad Nielsen’s large North Sámi dictionary [29–31] mentions similar names (in his spelling: *hai-suoinek, hai(sâ)-suoinek, njālgâ-hai-suoinek*) from Guovdageaidnu/Kautokeino and Kárášjohka/Karasjok ([30]: 288, 290), with *hāggjâsuoinek* as a deviant form. They are not identified, whereas the recent dictionary of Kåven and others translate *háisuoidni* and *háisǫsuoidni* into Norwegian *marigress* (= *Hierochloë odorata*), though this may be generalising ([32]: 241).

I have translated *háissa-* simply as scent, though some would argue that it should rather be translated as “strongly scented” or even “stinking”. The latter, however, does not fit with the frequent use of such grasses as perfume on account of their fine scent. Plants that are considered to be foul-smelling may receive other qualifying adjectives in Sámi, e.g. *guohca-* (“stinking, rotten”) in the widespread *guohcarássi* name for *Rhododendron tomentosum* (Stokes) Harmaja [33].

In addition to North Sámi, three other Sámi languages have been spoken in Norway (South, Lule and Skolt Sámi), but only by very small minorities. Within Norway, they are largely unexplored in terms of plant names and ethnobotanical traditions. The list of Sámi plant names compiled by Just Knut Qvigstad in 1901 [4] covers all the Sámi languages, but offers nothing in terms of names for scented grasses outside the North Sámi domain. It would not necessarily be helpful anyhow, since both this list and most dictionaries available [e.g., 29, 30, 31, 32, 34 – North Sámi; 35, 36, 37, 38 – South Sámi, 39, 40 – Lule Sámi, 41, 42, 43 – Inari Sámi, 44 – East Sámi] basically comprise lists of Sámi terms. Even when equivalents to “scented grasses” or “pleasant grasses” are included, they are rarely identified in terms of which plant species they belong to. As noted by Qvigstad, the same terms may have been used for other fragrant plants, e.g. *Achillea millefolium* L. in Troms, North Norway [(4): 305]. Qvigstad also collected material for a dictionary of the Sámi vocabulary used in southern Troms and northernmost Nordland [45], but no terms related to scented grasses are included there.

An updated dictionary of the South Sámi language has recently been compiled [36, 37], covering both Norway and Sweden. It does include some grass terms, but none related to scented grasses. Available dictionaries of the Lule Sámi language also fail to record terms related to scented grasses [39, 40]. The Inari Sámi dictionary compiled by Erkki Itkonen mentions *njalgasyejni* or “scent grass”, ([42]: 207), probably for an (unidentified) grass species, *syejni* usually referring to graminoids. T.I. Itkonen’s large dictionary of the Skolt and other East (excluding Inari) Sámi languages mentions χ*ājărāşş*^*E*^ from the Notosero area of Russia. It may have been used for scented grasses, but no identification is provided ([44]: 35). Veinö Tanner’s large ethnographic study of the Skolt Sámi population of the Finnish-Norwegian-Russian border area contains some notes on plant uses, but do not mention scented grasses [46].

#### Finnish

I have only recorded two different names in Norway, *hajuheinä* (“scent grass”) and *hyvähajuheinä* (“fine scent grass”), both well-known in Finnish-speaking communities in the Skallelv area of the Varanger peninsula (cf. [47–49]). The former is also used further west, at Vestre Jakobselv ([48]: 8). In addition, Solgunn Hesjevoll mentions *haisuheinät* (“scent grasses”) from the Børselv area of Porsanger, Finnmark ([49]: 99). Just as with the Sámi *háissa-* or *háisǫ-, haisu* may alternatively be translated as “strongly scented” or “stinking”.

### Finding, collecting and storing

Grasses may be difficult to identify, even to botanists. In folk tradition, scented grasses are usually collected in traditional sites. The exact spot may be located by the strong coumarin smell, as suggested by some of my informants. At Arnøya in Skjervøy, Troms, an old harvesting site at the island’s east side was described as follows: “It was an old woman who said, after midday, at four or five o’clock, then you can walk along the road, and you will notice the scent” (EBATA 2006:45). At Nordreisa in Troms, “there were some places, there was such an extra fine smell. We called it [the grass] *godluktgress*.” (EBATA 2006:88). At Seglvik in Kvænangen (Troms), I was told the approximate location of a harvesting site; the scent was considered sufficient to find the exact spot: “There you will smell something, there is such a fine scent.” (EBATA 1995:5). At Burfjord, also in Kvænangen, *Anthoxanthum nipponicum* was located by its smell: “At this natural meadow, the girls used their nose to find a kind of grass straw, which they thought had the finest scent. It grew among the stones.” (EBATA 2012:7). At Syltefjord in Båtsfjord, Finnmark, an old Norwegian female knew the smell, but provided no details of what the plant looked like: “I only know that it was outside the fence. We only noticed that there was such a very fine scent” (EBATA 2006:117).

*Hierochloë odorata* may form large stands, and predominate on damp meadows. Picking the right leaves may still involve difficulties, as other grasses may grow intermingled with it. Several Sámi informants had noted a detail that was useful for identifying *Hierochloë*: the basal part of the shoot has a reddish colour, as mentioned at Kunes in Lebesby: “Mostly, you were guided by the smell. You noticed the scent, now you were there.” The colour was of secondary importance: “Yes, the base is red. And there is almost like a red tinge at the nodes.” (EBATA 2015:24). A female from Porsanger also mentioned that the leaves were shiny: “When I walk along [the river] Stabburselva, then I collect, then I smell; [it is] very difficult to find, not many are able to. When they are fresh, the leaves are kind of shiny, and the stem is red.” She had no knowledge of the culms or inflorescence, which clearly passed unnoticed: “But I have not seen such straws.” (EBATA 2001:4). Again talking of a site at the Stabburselva river in Porsanger, a second informant told me: “And I know how it looks. I look for that red colour. And there is a red root, because you pull it up.” Despite pulling it up, she expressed concern that collecting material in this way was harmful; she considered it better to use a knife or a pair of scissors to cut the leaves, to avoid uprooting the plant (EBATA 2006:23).

Two Sámi females from Unjárga/Nesseby also pointed out that the red shoot bases were useful for picking out the correct material. The first, who happened to come walking by me in Bergebydalen, carrying a bundle of fresh leaves collected for making braids, commented: “It is just this, that they are red.” (EBATA 2007:70). The other woman had no experience of actually collecting material, but knew what to look for: “So I have tried to find it [without success], but I know that the stem should be red.” (EBATA 2007:72).

The red stem base was also known to a female Finnish informant from Skallnes in Vadsø, Finnmark; she mentioned the rather broad leaves as a second, characteristic feature (EBATA 2006:112). The broad leaves were pointed out as a prime character for identifying material by a male Finnish informant at nearby Skallelv: “It is very easy to pick out. When it is fully developed, it is this broad” [about 0.5 cm, shown by the fingers] (EBATA 2006:113). Another Finnish-speaking informant from the same area mentioned the long leaves in particular: “Yes, long leaves.” (EBATA 2006:115).

A Norwegian informant from Ekkerøy in Vadsø, Finnmark noted that the leaves were rather “coarse”; they are certainly more robust than in many other grass species. Again, the sweet scent was the main factor for finding the plant: “Yes, it was … one used the smell. But when one got used it, you picked it out from other grasses [by visual characteristics].” “To me, it was so coarse when you touched it. And it had that fine scent. So I think that is how we identified it.” (EBATA 2006:118).

These characters are mostly useful for selecting the right kind of material. In practice, people often relied on visiting traditional collecting grounds, where suitable material was known to occur, often abundantly, e.g. in Unjárga/Nesseby: “Yes, it was [these] customary places, where you go [to collect it]” (EBATA 2007:66). At least some preferred to keep these sites secret, though some stations are revealed by toponyms. Njálgsuoidnerohci (“pleasant-grass-hollow” in Unjárga/Nesseby has obviously been named based on a local occurrence of suitable material (EBATA 2007:71).

Others knew the ecology well enough to identify suitable habitats, as in Finnish tradition at Golnes and Skallelv in Vadsø, Finnmark; the habitats noted support the identification as *Hierochloë odorata*: “You find it up in the mire [i.e., in damp sites]. You don’t find it elsewhere” (EBATA 2006:111); “It grew along brooks” (EBATA 2006:106).

In Sámi and Finnish tradition, the leaves were often braided. Such braids are first mentioned by James Backhouse, an English Quaker missionary and botanist who travelled through northern Norway in 1853 and 1860. He identified it as deriving from *Hierochloë* . In his diary for 7 August 1860, while walking through the mountains at Langfjorden in Alta, Finnmark, he noted that “There was a good deal of Hierochloe borealis*”, adding in a footnote: “*This fragrant grass is platted by the Lapps into things like whip lashes, & sold as a perfume.” (cited from [50]: 44).

*Hierochloë* braids are known from many sites in Finnmark, including Alta (EBATA 2015:13, 2015:20), Guovdageaidnu/Kautokeino (EBATA 2006:23, 2006:43, 2006:126, 2015:3); Kvalsund (EBATA 2006:82, 2006:92), Lebesby (EBATA 2015:24), and Unjárga/Nesseby ([25]: 66; [51]: 62; EBATA 2007:67, 2007:69, 2007:70, 2007:71, 2007:72) – all North Sámi, and Neiden in Sør-Varanger (EBATA 2005:79, see Fig. 2) – Skolt Sámi. A couple of comments may be added: “I don’t know how they stored it, it was just braided …” (EBATA 2006:23); “Yes, of course they braided it, when it was dry, and hung it up.” (EBATA 2006:92); “The grass was collected and braided – some still do it” ([51]: 62). Olav Johansen provides an account from Deatnu/Tana: “Here at Vesterbukt there is a grass species which exudes a fine perfume scent when it is harvested at the right time. After collecting, the grass was braided and dried.” ([7]: 58). An old Sámi female from Guovdageaidnu/Kautokeino noted that braids should preferably be made from freshly collected material (EBATA 2006:87).

Braids were also well known in Finnish tradition, e.g. at Leirpollen in Porsanger, as noted by a female born in 1918: “I also have to tell you about *luktegresset* [the scent grass]. We collected *sennagress* which had such a fine scent. We called it *godluktsenna.* This we braided and hung up to dry.” ([52]: 17). Braids were also made at Børselv in Porsanger ([49]: 99, [53]), at Vestre Jakobselv in Vadsø ([48]: 8), and they were known by several informants at Skallelv in Vadsø: “Yes, it was braided” (EBATA 2006:10); “they picked it, and then they made such braids” (EBATA 2006:106); “it was braided” (EBATA 2006:113). At least some Norwegians had acquired similar tradition, e.g. in Pasvik, Sør-Varanger (EBATA 2015:27).

The braids vary considerably in their degree of perfection. Some make them rather roughly (e.g. [53]), while others put great care into their production, turning them into objects of considerable beauty. In some areas, the braids are made into small wreaths ([49]: 66, [51]: 61, [54]: 44). This was the case at Kunes in Lebesby: «It was made into, if not exactly braids, a kind of wheel. You braided it, and then you made it into a ring.” (EBATA 2015:24), and in Deatnu/Tana: “Braided such a wreath.” (EBATA 2006:13). Although the braids are decorative, the main purpose of braiding is obviously to protect the grass from breaking into fragments when drying.

Braids of *Hierochloë* are still sometimes offered for sale in Finnmark, e.g. in Guovdageaidnu/Kautokeino in 2006 (EBATA 2006:126), and at the summer market at Lakselv in Porsanger during recent years (EBATA 2006:23). In both these cases, the sellers were Sámi females involved in traditional reindeer herding. A female Sámi in Unjárga/Nesseby, interviewed in 2007, had just collected fresh material for her daughter, who was making braids for sale at the forthcoming market at Varangerbotn (EBATA 2007:70). In the two latter cases, the price asked for a braid was 50 Norwegian kroner (NOK).

In some areas, people did not make braids. In terms of scent, this hardly makes any difference. So far, informants of Norwegian ethnic origin have usually mentioned straight-forward bundles of material, e.g. at Olderfjord in Porsanger: “When you mention it, I remember my mother had it, such fine scent [grass]. She dried it. In a jar, one such [bundle]. And it dries. In the kitchen.” (EBATA 2006:93). The handling at Ekkerøya in Vadsø was equally simple:‟We did not make braids” (EBATA 2006:118). In other cases, the reason for not making braids was probably that the species collected was not *Hierochloë odorata*, but *Anthoxanthum nipponicum,* and thus hardly suited for braiding*:* “No, we just picked a few, and put it in the pocket or …” (EBATA 2006:89).

Dried material of *Hierochloë odorata* stores well. It will keep the fine scent for several years, as noted by some informants, e.g. in Finnish tradition: “Yes, I have collected it today. But it will retain its scents for several years. Look, this is [a bundle from] the year before last, and this one is from last year.” Commenting on the fresh bundle: “This one is still raw.” (EBATA 2006:113; cf. [47]: 49).

Similar use of *Anthoxanthum* spp. seems to have been rather rare. It has been confirmed from two areas in Troms, in Nordreisa and Kvænangen. The last record is by far the most detailed, and identification was confirmed both by a voucher specimen and the informant’s description of the plant material, noting that the material collected consisted of straws (i.e., the entire plant), and not leaves, which in *Anthoxanthum* are too small and short to allow use in braids:

“My aunt, she is 90 years old. When she was a child, they visited the field, natural meadows, and among all the straws they found growing there, they picked [this species], and dried it. And they made braids from it.” (EBATA 2010:47).

When the braids get old, the scent may be revived by moistening the dried-out material.

### Uses

The major uses of scented grasses are related to their fine scent, either directly, as perfume, or when stored with clothes. In the latter case, the insect-repellant effect has obviously also been an important factor.

#### Perfume

In the north, scented grasses, mainly *Hierochlo*ë *odorata*, but sometimes also *Anthoxanthum nipponicum* and *Milium effusum*, have been frequently used as a kind of perfume, both in Norwegian, Sámi, and Finnish tradition. The following records refer to *Hierochloë odorata*, as shown either by voucher specimens or the fact that the leaves were braided.

In the tiny Sámi settlement of Láhpoluoppal (in Guovdageaidnu/Kautokeino, Finnmark), this was the only available kind of perfume: “They didn’t use anything else for perfume.” (EBATA 2006:23). Such use was mostly restricted to finer occasions, e.g. the Easter festival and weddings – often combined in Guovdageaidnu/Kautokeino. They were not for everyday use: “No, they are only taken out at Easter time, when people left for the festival. Then, the scarf was procured. And there was such a fine smell.” “They did not embellish themselves much, only for Easter and weddings” (EBATA 2006:23).

Others used *Hierochloë* material as perfume when they were attending church, e.g. at Kvalsund in Finnmark:‟Yes, they placed that fine scent grass among the clothes, the fine clothes, which were used. It was perfume. I heard they talked about it. The fine clothes, when they were attending sermon.” In this case, it was not braided: “No, they just placed that grass in the clothes” (EBATA 2006:82). At Kunes in Lebesby, the same tradition was known: “And perhaps it was a kind of perfume, in *kofta* [the traditional Sámi garment], because of its fine smell. And it was used when going to church, in clothes. That is when it was used.” (EBATA 2015:24). Among the Varanger Sámi, scented grasses were well known and frequently used, as shown by several records from Unjárga/Nesseby: “It was common, they placed it in fine clothes, stored it with fine clothes. And they used in their caps.” (EBATA 2007:66). “The women used to have in their cap, so that it got a fine scent. And inside *kofta*” [the traditional Sámi garment]. (EBATA 2007:66). Øystein Nilsen provides an account of traditional use as perfume among the Varanger Sámi:

“When the grasses are full-grown in July, the scents of nature differ from those earlier in summer. The best scent is that of *marigresset.* The Sámi call it *njálggasuoidne,* godluktgress in Norwegian. It has a very pleasant scent, and previously, the Sámi used it as perfume. The girls placed it inside their kerchief or on the chest. The boys had in the fold of their headgear or in a pocket on the chest.” ([51]: 62).

Less detailed notes are available from some other sites in Finnmark, e,g. at Alta: “And then I got a braid of *godluktgress*, it has such a strong scent. And in Sámi, she called it *haisenna.* And then she told me that the females used to have it in their scarfs.” (EBATA 1998:3); “They braided it and placed it inside fringed kerchiefs” (EBATA 2015:20); “*Luktegress.* Yes, I know, the females often had it, more or less as a kind of perfume” (EBATA 2015:21). Similar records are available from stations further east: “Yes, they used it (…) [as perfume]. It was a fine scent.” (EBATA 2006:92; Kokelv in Kvalsund); “the women use is as perfume” (EBATA 1992:11, Kárášjohka/Karasjok). A record from Repvåg in Nordkapp probably refers to *Hierochloë* in terms of the Sámi used mentioned, although the local Norwegians collected *Milium effusum* for the same purpose: “The Sámi used to make braids from this grass and place it inside scarfs as perfume” (EBATA 2005:75) . In Unjárga/Nesseby, braids of scented grass were carried inside the dress or in the cap due to the fine scent ([25]: 66). In Deatnu/Tana, scented grasses may have been used as perfume by the females only; males should supposedly rather smell of sweat and tobacco ([7]: 58).

Similar use is known from Finnish tradition, at Vestre Jakobselv, Golnes and Skallelv in Vadsø. To serve as perfume, *Hierochloë* foliage was usually braided, and placed e.g. in male headgear: “The men usually had *godluktgress* in the hat!” ([48]: 8). “Yes, under the cap.”They used to braid it, in the old days, and then they placed it under the cap.” “Those [leaves] that my father and others put beneath the cap.” (EBATA 2006:111); “*Hajuheinä* … my father and grandfather, they used to braid it, and put it inside [the cap]”. (EBATA 2006:112); “(…) which one used in the caps to get a fine scent. One made a ring and placed it inside.” (EBATA 2006:10); “Older boys used it in their caps.” (EBATA 2006:106); “There were many who used it inside the sixpence caps. It was braided.” (EBATA 2006:113); “And I remember my uncle, he used it in his cap, the sixpence.” (EBATA 2006:115). Yet another informant noted that the grass was braided, and that his father had used beneath his cap (EBATA 2006:114).). It might also be useful for romantic occasions, as noted by a man from Børselv in Porsanger, who “says he used it when he was visiting his loved one” ([49]: 99). In recent years, people at Vækker, also in Porsanger, have tried to use the grass to produce perfume in a more traditional sense of the word, if only on a small scale ([49]: 99). Some kind of scented grass was also known to a Finnish-speaking woman interviewed at Brennelv in Porsanger, but the interviewer failed to record what is was used for, recording only that: “Yes, I have collected it and used it.” ([55]: 34). Use as perfume was also noted in the ethnically mixed settlements at Lakselv in Porsanger: “Previously, *godluktgress* was frequently used by the women. When they embellished themselves for parties or finer visits, they placed *godluktgress* in the fold of their kerchief.” ([57]: 105). Further east, the Finnish inhabitants of the Vestre Jakobselv village in Vadsø collected *Hierochloë odorata* for perfume and other purposes: “The grass had a fine scent, and was also used as perfume. One made it into small braids which were placed beneath the clothes.” (48]: 8). Accordingly, *hajuheinä* or “scented grass” is noted among the more important local plant resources ([48]: 8–9; cf. [56]: 254).

At Burfjord in Kvænangen (Troms), *Anthoxanthum nipponicum* was used in the same way and for the same purpose, although it is not really suited for making braids: “They made a braid from it, and dried it. Afterwards, it was used to provide a fine scent in pockets and kerchiefs when they were going to church, to religious congregations, and whenever they wanted to embellish themselves.” (EBATA 2012:7). Based on the description given, *A. nipponicum* seems to have been the source of the scented grass collected at Snefjord in Måsøy, Finnmark. It was used in much the same way: “In the past, the males used to have it in their pocket. Previously, they did not have [commercial] perfume, so the males” [used it] (EBATA 2006:89).

Other records are unidentified in terms of the species used; most of these probably refer to *Hierochloë odorata.* At Arnøya in Skjervøy, Troms, I was given the following account of local Norwegian use: “*Luktegras,* an old tradition, it was to place it in [the clothes]. (..) During church weekends, the head scarf should have a fine scent. And that was when one collected *luktegras* and placed it in the scarf, [or] beneath the pillow.” (…) “It was to get perfume” (EBATA 2005:45).

At least nowadays, some collect scented grasses and make braids partly for decorative purposes, e.g. in Unjárga/Nesseby: “I have it as a decoration. I keep it for weeks, so that it makes a fine scent.” (…) “I learned it from my aunt. She used to have it on the wall.” (EBATA 2007:69). Even in the past, some kept braids for the fine smell only: “It was mainly for the scent.” (EBATA 2007:66). Similar use has been noted among the Finnish-speaking inhabitants at Børselv in Porsanger: “According to a person from Vækker, it was dried and hung up in the house”.

#### Storing with clothes

A frequently encountered use of scented grasses is for storing with clothes, as noted in numerous records from Finnmark, including Alta (EBATA 2015:13, 2015:20), Guovdageaidnu/Kautokeino ([27]: 518), Kvalsund (EBATA 2006:82, 2006:92), Lebesby (EBATA 2015:24), Porsanger ([57]: 105, EBATA 2001:4), Deatnu/Tana (EBATA 2006:2), Unjárga/Nesseby (EBATA 2007:66, 2007:71; ([58]: 232], and Sør-Varanger (EBATA 2015:27). This obviously served a double purpose, providing the clothes with a sweet, fine scent, but also keeping moths and other insects away. Again, *Hierochloë odorata*, often braided, was the main material used, e.g. in Guovdageaidnu/Kautokeino: “And then, this *háisuoinnit* …” “Yes, they used it, collected, and put it in silk scarfs, fine clothes, that it should have a nice scent. And also in chests.” (EBATA 2006:66), and in Kvalsund: “They just placed that grass among the clothes.” (EBATA 2006:82). At Kokelv in Kvalsund, an old woman noted that it was used with fine clothes, and that the grass had to be dry before it was placed between them, “so dry that it did not spoil them” (EBATA 2006:92).

Similar records are available from Sámi settlements further east in Finnmark, e.g. at Porsanger: “Previously, they used it a lot, placed it in the suitcase with clothes, so that they should get a fine scent. And it was a fine scent” (EBATA 2001:4). At Kunes in Lebesby: “Well, it was used in the chest for storing clothes, that is where it was used. Due to its fine smell.” (EBATA 2015:24). In Deatnu/Tana, it was “placed inside the clothes, inside cupboards, in chests, previously.” (EBATA 1996:2); “and also in clothes, when one stored clothes” (EBATA 2006:13). A female Sámi from Unjárga/Nesseby recalled her mother’s use of *njálggsuoidni:* “Yes, she used it, for we always had it in the drawer of the night table (…), and between clothes, and in [other] drawers.” (EBATA 2007:71). Olav Johansen provides an account from Deatnu/Tana: “The houses then were small. For that reason, one did not have room for cupboards. The fine clothes were stored in a chest. When the braids of *godluktgress* were placed together with the clothes, the scent spilled over to them.” ([7]: 58).

Similar use is known from Finnish tradition in Finnmark. At Leirpollen in Porsanger, a female informant noted that braids were made, “and afterwards we could for example place it in the cupboard and give the clothes a pleasant scent. It was the kind of perfume we had.” ([52]: 17). At Børselv in Porsanger, another informant noted that the grass “was braided and used among the clothes” ([49]: 99). It was put to similar use further east, e.g. at Skallnes in Vadsø: “And my mother used to have it in the drawer, so that the clothes should get a fine scent.” (EBATA 2006:112). In the nearby Skallelv village, one informant referred to his mother using it in the drawer (EBATA 2006:114). Another provided a more detailed account: “Yes, you know, previously, in the old days, they used to place it – they did not have cupboards for clothes” – and thus it was stored with them, in chests or otherwise (EBATA 2006:113).

In the high north of Norway, people of Norwegian ethnic origin also stored *Hierochloë odorata* with their clothes, e.g. at Båtsfjord in Finnmark: “We called it *godluktgress.* I remember we had it at home. My grandma used to go and pick it.” “She used to place it in the drawer, so that the clothes should have a fine scent. At that time, they did not buy perfume. It was at Syltefjord.” (EBATA 2006:117). Tradition in Sør-Varanger, close to the Russian border, was the same: “I know that *luktegress* was braided and placed in the wardrobe” (EBATA 2015:27). A record from the ethnically mixed Lakselv area of Porsanger may also be included here: “The grass was placed in the chest of drawers, between the clothes” ([57]: 105). Two further records confirm such use in Norwegian tradition. Both are likely to refer to *Hierochloë odorata,* e.g. in Alta: “My mother had heard of it, but could not say much more. She called it *duftgress*. She said it was something they used to put in the drawers with linen clothes.” “It was something that was collected, dried and placed among the linen.” (EBATA 2006:6).

Ove Arbo Høeg provides a rather similar record of using *Anthoxanthum* from Engerdal in Hedmark, SE Norway, where “it was collected in small braids or bundles and placed among the kerchiefs in the chest drawer. Very fine scent. Was usually braided with three cords” ([6]: 224). This tradition is still remembered, according to a recent record: “I know this from Engerdal, from my own childhood as well.” (EBATA 2015:6). According to my informant, *luftgrasbonk* was *Anthoxanthum nipponicum,* “for the grass was collected at pastures up in the mountains. These plants had the finest scent. They were collected in small braids, and as far as I know, placed among kerchiefs in the drawers to provide a fine fragrance”. *Hierochloë odorata* is uncommon in the area, and thus difficult to obtain (EBATA 2015:6). This may, thus, be the rare exception where *Anthoxanthum* has been braided, or sort of braided, even though only the short culms provide sufficient material for doing so (cf. the “three cords” noted above), as shown by an additional comment on the Engerdal tradition: “Yes, the straws are braided, although this is perhaps exaggerating a bit, but several straws were strung together in a braid-like way, probably to increase the fragrance.” (EBATA 2015:7).

#### In houses

Scented grasses could also be used in houses, at least when they were still partly built from turf, as suggested by a record from Deatnu/Tana: “I think it was used in the *gamme* [turf house] previously.” (EBATA 2006:13). Øystein Nilsen mention such use from Unjárga/Nesseby: “The grass braids may be hung at various places in the house. They will keep for several years. A stench of water will strengthen the smell.” ([51]: 62).

People who had access to abundant material sometimes used *Hierochloë odorata* at toilets, to provide a fine scent and camouflage whatever malodours might occur, e.g. in Sámi tradition at Heammonjávvi in Guovdageaidnu/Kautokeino: “[You] can also place them at the toilet, they are good when they dry.” (EBATA 1996:10). An old Sámi female from Kokelv in Kvalsund commented on this practice, noting that they could not spare material for such luxury – depending on local availability: “Yes, it is possible, that [at some places] there was such a surplus of it, but we did not have [enough]” (EBATA 2006:92). In Deatnu/Tana, drying braids ensured a good scent in all parts of the house: “Then, many braids were hanging inside to dry. These braids may have had the same function as a deodoriser which people now have hanging in the toilet” ([7]: 58). *H. odorata* is still used for this purpose at Seiland (Hakkstabben) in Alta (EBATA 2015:14).

The Finnish minority likewise used *Hierochloë* to provide their homes with a pleasant odour: “And some put it in the bedclothes, and some put it in the bed. Such a fine, fresh smell.” Commenting on an old braid: “It is many years old, but there was a fine scent from it. Many placed it everywhere.” (EBATA 2006:106).

People of Norwegian ethnic origin also sometimes used *Hierochloë odorata* to provide their houses with a pleasant scent, e.g. at Olderfjord in Porsanger (EBATA 2006:93, cited above). An 88 year old man from Ekkerøy in Vadsø provided some further details: “Yes, we used it a lot.” The leaves were made into simple bundles: “One made them into bundles, and then we had it hanging inside the house, to get a special scent. It had a fine scent.” (EBATA 2006:118). At Steinkjer in Nord-Trøndelag, central Norway, bundles of *Anthoxanthum* and *Hierochloë* were hung on the wall to provide a fine scent ([6]: 224).

#### Isolation

Scented grass, obviously the long and flexible leaves of *Hierochloë odorata*, has also found some use as isolation material. According to an old Sámi female from Guovdageaidnu/Kautokeino, still moving with her reindeer herd between the interior plateau and the coast in the traditional way, *háisuoinnit* (in this case *Hierochloë odorata* ssp. *odorata*) could be used to insulate shoes and clothes much in the same way as various sedges (*Carex* spp.) are commonly used. In her opinion, *háisuoinnit* were warmer than the sedges: “And then inside skin clothes, it is much warmer than *gamassuoidni* [‘shoe grass’. i.e., *Carex* spp.]” And they are so soft, the leaves hang like this …” (EBATA 2006:66). Similar use is also known from Deatnu/Tana: “The only thing my father could remember (…) was that some also used it together with sedges in *komager*”, i.e. in traditional skin shoes (EBATA 2006:28, 2006:31).

#### Folk medicine

In all likelihood, the scented grass mentioned in Adolf Steen’s compilation of Sámi folk medicine [8] was *Hierochloë odorata,* and not a *Carex* species as he was lead to believe by the fact that both are regarded as *suoidni* (plural *suoinnit*) or a “graminoid” in Sámi tradition. It was used as insulation material, but could also provide relief for frozen fingers:

“When toes or fingers are frozen, they are covered with hāggjâ-suoinek [well-scented grass]. (godlukt-gress). There are two kinds of *sennegress:* The common sedge which is used in footwear during both summer and winter, and a finer sort, with a fine smell, which is used in mittens made of [reindeer] skin. It is the last-mentioned kind which is called hāggjâ-suoinek.” ([8]: 40).

No source is given, but Steen had probably gained knowledge of the scented grass tradition during his residence in Guovdageaidnu/Kautokeino. He included an almost similar paragraph in a Norwegian children’s book published the same year, describing the life of reindeer herders based at Guovdageaidnu/Kautokeino:

“There are two kinds of *senner*. It it *akko* [in modern North Sámi: *áhkku*, “grandma”] who tells us so. It is the common sedge which is used in both winter and summer footwear, and then a finer sort which is called *godluktsenner* [Norwegian: well-scented *senner*]. In winter, the Sámi use a kind of large skin mittens called *gistak.* Inside these, they have *godluktsenner* instead of woolen mittens.” ([59]: 52–53).

### Taste

Children have frequently extracted the culms of various grass species from the nodes and chewed at the white, interior part. At least one record mentions such chewing of scented grasses, among the Sámi at Vestertana/Deanodat in Deatnu/Tana: “It is good to chew” (EBATA 1996:2). The local name for it, *njálggaháisuoidni,* may thus be interpreted both as meaning “pleasant scent grass”, or refer to the alternative meaning of *njálgga,* “sweet” or “well tasting”.

In the interior part of SE Norway, *Anthoxanthum* was previously collected in small bundles, dried and used as basis for a drink, called *fjell-te* (“mountain tea”). It was boiled for about ten minutes, whereupon sugar was added ([6]: 224, [60]: 94).

## Discussion

Tradition related to scented grasses, in particular *Hierochloë*, is widely distributed in the northern hemisphere. The grasses are well known in neighbouring Finland and Sweden as well, especially in the north. The Finnish vernacular names recorded in Norway are also used in Finland [61].

Margareta Svahn’s treatise on grass names in Scandinavia comments briefly on names for sweet-smelling grasses, mainly in Sweden, but says nothing on their identity ([62]: 50). In Swedish tradition, scented grasses are known as *luktgräs* and *goluktgräs*, which are Swedish parallels to the predominant Norwegian names. According to Ingvar Svanberg [63], the names are mostly used for *Anthoxanthum odoratum* and *Hierochloë odorata*, more rarely *Milium effusum* – in other words, the same three genera utilized in Norway. They may also be referred to as *myskgräs.* People in the northern parts of Sweden used such grasses as nosegays when going to church. In this area, people also frequently made braids, which were put in the clothes to provide them with a fine scent. Braids were stored with clothes, and some believed that they kept moth away ([63, 64]: 148, [65]: 167). In Härjedalen, bundles of *Anthoxanthum* could be seen hanging above the bed ([66]: 719). Less frequently, scented grasses were mixed with tobacco to provide a finer taste, e.g. in snuff.

Linnaeus [67, 68] encountered a living tradition related to scented grasses during his visit to the northern parts of Sweden in 1732. The entry for *Hierochloë* (No. 53) in his *Flora Lapponica* merely notes that “its fine scent could compete with that of the above mentioned *Milium*” ([68]: 41). He identified the (main) species used as *Milium effusum* (No. 35), noting that “the Sámi girls use to collect a bundle of the leaves of this grass while they are herding the reindeer, which they place in the bag hanging at their lower belly, where they store their tobacco. However, I do not know, if they, by doing so, aim mostly at providing the tobacco or themselves with a pleasant scent.” ([68]: 37). He may have been generalising based on a single observation of *Milium effusum* leaves used for this purpose; in all probability, *Hierochloë odorata* was the preferred and most frequently collected material by the Sámi in Sweden as in Norway. Within the area of the South and Lule Sámi languages, the use of scented grasses as perfume may to some extent have been replaced by the fragrant fungus *Haploporus odorus* (Sommerf.) Bond. & Sing. It was eagerly sought for in Sweden, both by the Sámi and ethnic Swedes, and used both as perfume and to flavour food, e.g. bread and pancakes ([9]: 282ff), [69]: 42). On the Norwegian side of the border, the species is very rare, and only known from a few, widely dispersed stations [70]. It is much more widely distributed, and thus accessible, in Sweden and Finland [71].

Further south, Danish tradition related to scented grasses is very sparse. Carl Gottlob Rafn noted in 1796 that *Anthoxanthum odoratum* was placed in tobacco containers to provide a fine aroma ([72]: 425). According to Vagn J. Brøndegaard, this species was sometimes cultivated on meagre soils to provide hay with a fine scent and taste ([73]: 132) – mistakenly in terms of taste, for *Anthoxanthum* has a bitter, unpleasant taste, and is listed among the poisonous grasses of North America ([14]: 908–910).

The use of scented grasses as perfume when going to church has old, European roots. The Latin name *Hierochloë* means “holy grass”, and old sources mention that it was strewn outside church on feast days, e.g. in Germany ([74]: 867). According to David J. Mabberley, *H. odorata* was introduced to Scotland by Prussian monks, and cultivated there for the same purpose ([23]: 52). Of its few Scottish localities, many are close to ecclesiastical sites ([75]: 17).

The so-called bison grass or *żubrówka* of Poland is *Hierochloë odorata,* cf. Polish *zubr, “*bison”, or rather the European wisent (*Bison bonasus* (L.); the same name motif is found in Lithuania ([76]: 167). The grass is used as the colouring and flavouring agent of an eponymous vodka [77], but no ethnobotanical account of its history seems to be available. Similar, *Hierochloë-*flavoured vodkas are also made in Russia and the Republic of Belarus (Aleksei Kravchenko, pers. comm.). According to local lore, or at least the *żubrówka* producer’s campaign material, the bison is supposed to gain its strength from eating *Hierochloë*, and the plant may impart similar virtues, strength and virility, in humans. Pauline Javani provides some notes on the drink and its history [78]. According to Polish historians, vodka was a Polish invention, dating back to 1405 ([78]: 90), but the Russians may contest this. Thus, the *żubrówka* drink cannot be older. Other sources, however, suggest a long and still extant Polish tradition of harvesting *Hierochloë.* Seeds of *Hierochloë* cf. *australis* (Schrad.) Roem. & Schult. were retrieved in an archaeobotanical study of a Neolithic settlement in central Poland, which may point to early collection and use [79]. This species is threatened in present-day Poland, partly due to excessive collecting [80], and cultivation seems an obvious alternative to provide necessary material [81].

The lists of vernacular names for *Anthoxanthum odoratum* collected in the 19th century Russian empire by Nikolay I. Annenkov leaves no doubt that both the Russians and various subject peoples considered its scent as pleasant; other names refer to its yellow colour rather than is fragrance ([76]: 39). Russian vernacular names for *Hierochloë odorata* mostly refer to its bitter taste ([76]: 167). Annenkov also noted that *Anthoxanthum odoratum* was used to cure asthma and difficulty of breeding ([76]: 39). *Hierochloë odorata* remains a popular medicinal plant in Russia, and is used for many diseases, but is not included in the official Russian pharmacopoeia. It also finds use in food production, e.g. for salted herring and in some pastries (Aleksei Kravchenko, pers. comm.). Both *Anthoxanthum odoratum* and *Hierochloë odorata* have been and still are used as flavouring for tobacco ([76]: 39; Aleksei Kravchenko, pers. comm.). A special study is needed to clarify the uses of scented grasses among the many indigenous groups of Russia. Others are better qualified, in terms of the knowledge and language skills needed, to review the uses that may occur within this vast area, with almost 200 ethnic groups, and a multitude of languages belonging to widely different language families.

North American traditions related to scented grasses form an interesting parallel to the uses in Norway, demonstrating that very similar traditions have evolved independently among indigenous populations in America and Eurasia. *Hierochloë odorata* or sweetgrass has found a variety of uses among the native tribes of North America ([82], [83]: 266). There as well, people were taught to identify the plant based on its purple base – and the shiny leaves [84]. On account of its fine fragrance, it was used it as a kind of perfume by the Blackfoot [82, 85, 86], Cheyenne ([87]: 170), Kiowa ([88]: 15), Lakota ([89]: 49), Menominee ([90]: 75), Montana ([91]: 28), Okanagan-Colville ([92]: 55), Omaha ([93]: 323), and Thompson Indians ([94]: 503, [95]: 141–142), in the latter case tied in the hair or as an arm or neck ornament, or as a decoction to wash the hair and body ([94]: 476). The Blackfoot and Flathead likewise used the grass as a decoration in the women’s hair ([83]: 266, [91]: 28). Some tribes made braids from it, just as in Scandinavian tradition, e.g. the Blackfoot ([85]: 9, [96]: 51, [97]: 278), Cree and Métis ([98]: 292), and at least the Okangan-Colville stored such braids with their clothes in order to give them a nice smell ([92]: 55). Other tribes recognized their insecticide function, e.g. the Flathead ([83]: 266). The pleasant scent may also be involved in the Kiowas’ use of *H. odorata* as stuffing in mattresses and pillows ([88]: 15), though it should be noted that other tribes considered the grass to have apotropaic abilities. In Cheyenne tradition, the grass was burned to protect against thunder and lightning ([83]: 266), or to protect the home from evil ([99]: 10).

*Hierochloë odorata* figures boldly in the religious tradition of the Blackfoot Indians, who chewed the grass to promote endurance during their ceremonies, which frequently involved prolonged fasting. Grass bands were part of the headdress while sundancing ([85]: 9). As part of the ceremony, the grass was burned, e.g. on small altars, and prayers said ([86]: 20, [97]: 273–274). The smoke was supposed to purify the participants. Grass was also mixed with tobacco and ceremonially smoked ([91]: 28). In somewhat similar fashion, the Sioux and Cheyenne burned the grass to purify various ritual items used in their sundance ([87]: 170, [91]: 28, [99]: 9). In the tradition of the Lakota, guardian spirits were called upon by burning the grass ([89]: 49), and the Menominee burned it to honour their gods ([90]: 75). It was put to similar use in the NW boreal forests of Canada, both by Cree and Métis, who believed the smoke purified the attendants and carried prayers up to the creator ([98]: 292). The Kiowa included *H. odorata* in their peyote ceremonies, sprinkling dried grass leaves over fire ([88]: 15). It also found religious use among the Dakota ([100]: 359, [101]: 91), in more modern times also when attending church service ([100]: 21), and among the Omaha ([93]: 329), Pawnee and Ponca ([83]: 266–267). A chief of the Chippewa tribe mixed *Hierochloë* with tobacco intended for a peace pipe ([101]: 22).

Contrary to Scandinavian tradition, *H. odorata* was frequently used as a medicinal plant in North America, e.g. by the Blackfoot, who inhaled the smoke from burning leaves to cure colds ([86]: 20), and drank an infusion for coughing or sore throats ([85]: 72). In Cree tradition, a decoction was given to young girls to facilitate childbirth ([98]: 292).

Other uses, e.g. in basketry [82, 84], differ from those recorded in Norway. This is a tradition found among several North American tribes, including the Iroquois ([102]: 67), Malecite ([103]: 6), Menominee ([90]: 75), Micmac ([104]: 258), and Thompson Indians ([94]: 503). Locally, *Anthoxanthum odoratum* has also been used to make baskets, perhaps more on account of its fine scent than being really suitable in terms of providing lasting utensils. Such use is known among the Abnai ([105]: 175), Ojibwe ([106]: 419), and Potawatomi ([107]: 120).

Returning to Norway, it may be safely concluded that our use of scented grasses fits into a tradition that is widely distributed in the northern hemisphere – and here as well, *Hierochloë odorata* is the primary source of suitable material. In a Norwegian context, the use of scented grasses is somewhat unusual by being (with few exceptions) geographically restricted to a small part of the country, despite the fact that the plant material needed (*Anthoxanthum* spp.*, Hierochloë* spp. and *Milium effusum*) is available almost everywhere. In most cases, traditions related to useful plants in Norway are as widely distributed as the species themselves, as is evident e.g. for *Pinguicula vulgaris*, frequently used to curdle milk ([108]), and *Rhodiola rosea,* with a variety of uses ([109]). *Linnaea borealis* provides an example of a deviant pattern, almost complementary to that shown by the scented grasses [110]. It is an important medicinal plant in southern and central Norway, and generally neglected in the north, even if the species is common there as well, and presumably contains the same chemical compounds. Whereas this pattern has no obvious explanation, the distinctly northern distribution of the use of scented grasses has a likely one. Originally, it may have been more or less exclusively a Sámi tradition, widely distributed and well known in the main North Sámi area. Over time, it has been adopted by people of Norwegian and Finnish ethnic origin living in the same area. With the sole exception of Ove Arbo Høeg’s record from the Forsand area of Rogaland ([6]: 224), all records of similar use further south in Norway have been made in areas with (South) Sámi minorities, and could thus derive from their tradition. A map showing the geographical distribution of traditions related to *Rhododendron tomentosum* (Stokes) Harmaja in Norway [33] would have produced almost the same result, but only partly for the same reason. Again, Sámi tradition related to such use is much stronger than its Norwegian counterpart, but in this case, the species also has a similar distribution pattern, heavily concentrated to the main North Sámi areas.

At present, the tradition related to scented grasses is certainly much stronger within the North Sámi and Finnish ethnic minorities than among ethnic Norwegians. At least among the Sámi, it is still transferred to new generations, serving partly as an ethnic marker. As such, it may still enjoy a long life.

## Conclusion

Within Norway, traditions related to scented grasses are heavily concentrated to the North Sámi strongholds of northern Troms and Finnmark. Within this area again, all ethnic groups, including ethnic Norwegians and Finns, have named and collected such grasses, mainly *Hierochloë odorata.* They were used as perfume, for storing with clothes, to provide the house with a sweet scent, etc. Despite their importance here, scented grasses have been largely overlooked in previous Norwegian ethnobotanical literature, obviously because most authors lacked knowledge of the Sámi (and Finnish) languages, and the high north in general. The tradition’s distribution pattern suggests that this was originally a Sámi tradition, adopted by their Norwegian and Finnish neighbours. The uses and traditions recorded in Norway have clear parallels elsewhere in Europe, e.g. in Sweden, and among the indigenous tribes of North America; the latter suggesting that similar traditions may evolve independently wherever suitable, coumarin-scented source material (*Anthoxanthum* and *Hierochloë* species in particular) is found.
